# Prognostic utility of perineural invasion in individuals with rectal cancer receiving neoadjuvant chemoradiotherapy: a meta-analysis

**DOI:** 10.3389/fonc.2026.1845905

**Published:** 2026-09-03

**Authors:** Meiyu Chen, Haoyu Yuan, Chunfang Xu

**Affiliations:** 1 Department of Gastroenterology, The First Affiliated Hospital of Soochow University, Suzhou, Jiangsu, China; 2 Department of Orthopedics, The First Affiliated Hospital of Soochow University, Suzhou, Jiangsu, China

**Keywords:** meta-analysis, neoadjuvant chemoradiotherapy, perineural invasion, prognostic, rectal cancer

## Abstract

**Objective:**

This study aimed to systematically examine the prognostic role of post-neoadjuvant chemoradiotherapy (nCRT) perineural invasion (yPNI) in patients with locally advanced rectal cancer (LARC) treated with nCRT.

**Methods:**

This study was performed according to the Preferred Reporting Items for Systematic Reviews and Meta-Analyses (PRISMA) guidelines. PubMed, Embase, Cochrane Library, Web of Science, China National Knowledge Infrastructure (CNKI), Wanfang Data, VIP Chinese Science and Technology Journal Database (VIP), and Chinese Biomedical Literature Database (SinoMed) were searched to identify eligible studies. Cohort studies that satisfied the inclusion criteria were included, and multivariable-adjusted effect sizes were extracted. The Newcastle-Ottawa Scale (NOS) was used to evaluate the quality of studies. Meta-analyses were carried out using STATA 15.1.

**Results:**

A total of 36 studies involving 18,721 patients were included. The pooled prevalence of yPNI was 15.9%. Pooled results showed that yPNI was significantly associated with poorer overall survival (OS) (hazard ratio (HR) = 1.90, 95% confidence interval (CI): 1.63-2.21), recurrence-free survival (RFS) (HR = 1.96, 95% CI: 1.50-2.56), disease-free survival (DFS) (HR = 1.93, 95% CI: 1.55-2.42), cancer-specific survival (CSS) (HR = 1.98, 95% CI: 1.66-2.36), local recurrence-free survival (LRFS) (HR = 2.54, 95% CI: 1.86-3.48), and metastasis-free survival (MeFS) (HR = 2.82, 95% CI: 1.56-5.10). These findings suggested that yPNI-positive patients had a higher risk of recurrence and death. Sensitivity analyses further validated the robustness of the findings.

**Conclusion:**

yPNI was associated with adverse survival outcomes in patients with LARC treated with nCRT. Further studies are needed to determine whether yPNI provides additional prognostic information beyond established clinicopathological factors.

**Systematic Review Registration:**

https://www.crd.york.ac.uk/PROSPERO/view/CRD420251230519, identifier CRD420251230519.

## Introduction

Colorectal cancer (CRC) is the third most frequently diagnosed cancer and the second leading cause of cancer-associated death globally, after lung cancer (1). Currently, approximately one-third of individuals present with locally advanced rectal cancer (LARC) at initial diagnosis (2). LARC includes tumors that have grown through the muscularis propria and invaded the outermost layer of the rectum (T3), or have penetrated the rectal wall and may have invaded adjacent organs or structures (T4), and/or are accompanied by lymph node metastasis (N+) (3). At present, the standard treatment paradigm for LARC has shifted from traditional postoperative adjuvant therapy to a comprehensive treatment strategy combining preoperative neoadjuvant chemoradiotherapy (nCRT) with surgery, with total mesorectal excision (TME) as the core component (3, 4). The application of nCRT has markedly reduced local recurrence rates and improved tumor downstaging rates and sphincter preservation rates (5). Nevertheless, even after standardized full-course treatment, substantial heterogeneity in prognosis persists among patients, and some patients still experience poor survival rates and a high risk of distant metastasis (6, 7). Therefore, identifying biomarkers that can effectively predict patient prognosis is crucial for achieving individualized precision treatment, optimizing follow-up strategies, and exploring novel therapeutic targets.

Perineural invasion (PNI) refers to tumor cell invasion into the perineurium, endoneurium, or epineurium (8). PNI is an established adverse prognostic factor associated with aggressive tumor behavior, an increased risk of local recurrence, and poorer survival in several malignancies, including pancreatic cancer (9), prostate cancer (10), and esophageal cancer (11). Furthermore, previous studies have demonstrated the prognostic significance of PNI in patients with CRC who have not received neoadjuvant therapy (12, 13). However, the nCRT era poses new challenges to both the pathological assessment and prognostic interpretation of post-nCRT perineural invasion (yPNI). nCRT can induce significant histological alterations in tumors, such as fibrosis, necrosis, and inflammatory responses. Thus, it is increasingly complex and difficult to accurately identify residual tumor cells and their infiltration patterns, particularly yPNI, in postoperative pathological specimens (14, 15). Several retrospective studies have evaluated the prognostic significance of yPNI in surgical specimens, but their findings have been inconsistent. Some studies have reported that yPNI positivity remains significantly associated with poorer survival outcomes after multivariable adjustment (16, 17), whereas others have suggested that this association is attenuated or no longer statistically significant (18, 19).

A large-scale, high-quality systematic evaluation of the prognostic utility of yPNI in rectal cancer is lacking. Therefore, this systematic review and meta-analysis aimed to evaluate the association between yPNI and survival outcomes in patients with LARC treated with nCRT and surgery, and to examine the consistency of this association across study and model characteristics.

## Methods

This study followed the Preferred Reporting Items for Systematic Reviews and Meta-Analyses (PRISMA) guidelines (20). The protocol was registered with the International Prospective Register of Systematic Reviews (PROSPERO; registration number: CRD420251230519).

### Search strategy

PubMed, Embase, Cochrane Library, Web of Science, China National Knowledge Infrastructure (CNKI), Wanfang Data, VIP Chinese Science and Technology Journal Database (VIP), and Chinese Biomedical Literature Database (SinoMed) were searched from inception. The initial search was performed on September 22, 2025, and an additional search of Chinese databases was conducted on July 27, 2026. The search strategies combined controlled vocabulary terms (e.g., medical subject headings (MeSH) and Emtree terms, where applicable) with free-text terms. The main search concepts included rectal cancer and perineural invasion. Equivalent Chinese terms were applied for searches of Chinese databases. The detailed search strategies for all databases are provided in **Supplementary Appendix A1**. Moreover, reference lists of related systematic reviews were also screened to identify more eligible studies.

### Inclusion and exclusion criteria

Studies meeting the criteria below were included in this study: (1) Participants: individuals with LARC who underwent nCRT followed by surgical resection, with yPNI assessed on post-treatment surgical specimens, aged ≥18 years. PNI generally refers to tumor cells invading the nerve sheath or perineural space. yPNI specifically denotes PNI evaluated on surgical specimens after nCRT. (2) Exposure: individuals were stratified into positive and negative groups according to the status of yPNI. (3) Study design: cohort studies. (4) Outcomes: including overall survival (OS), recurrence-free survival (RFS), disease-free survival (DFS), cancer-specific survival (CSS), local recurrence-free survival (LRFS), and metastasis-free survival (MeFS). (5) Multivariable-adjusted hazard ratios (HRs) for survival analysis and their corresponding 95% confidence intervals (CIs) were either reported directly in the original studies or could be obtained by combining adjusted estimates reported for mutually exclusive subgroups.

Studies meeting any of the criteria below were excluded: (1) animal or cell experiments, case reports, study protocols, comments, letters, editorials, and conference proceedings; (2) publications with missing data or severe errors; (3) duplicate reports or reports with potentially overlapping cohorts. Potential overlap was assessed according to institutions, databases, and enrollment periods. When overlapping cohorts reported the same outcome, only one report was retained. Specifically, the report with the largest cohort was preferred. When sample sizes were similar, the most recent report was selected. If the reports were otherwise comparable, the report providing the most comprehensively adjusted effect estimate was retained. Reports with uncertain overlap were retained in the primary analysis but excluded from sensitivity analyses; (4) articles with full texts unavailable; (5) studies reporting only univariable HRs without multivariable adjustment.

### Literature screening and data acquisition

The searched records were imported into EndNote. Two researchers independently screened the titles and abstracts of the literature against the inclusion and exclusion criteria. Subsequently, the full texts of the potentially eligible studies were reviewed. Any divergences were addressed via discussion or by consulting a third researcher. Two researchers independently acquired the data from the finally included studies using Excel 2016. The acquired information included: first author, publication year, country, study design, treatment regimen, study center, follow-up details, neoadjuvant or adjuvant therapy, survival endpoint, type of survival analysis, multivariable model characteristics, HRs and their corresponding 95% CIs, and clinicopathological characteristics. Further details on outcome definitions and effect size extraction are provided in **Supplementary Appendix A2**.

### Quality assessment

The quality of the included studies was assessed using the Newcastle-Ottawa Scale (NOS) (21). NOS involved three domains, namely selection, comparability, and outcome, with scores varying between 0 and 9 stars. Studies with an NOS score ≥6 were deemed to be of high quality.

### Statistical analysis

The pooled HRs and their 95% CIs were computed to evaluate the prognostic significance of yPNI in individuals with LARC. Heterogeneity across the studies was assessed with the I^2^ statistic and the Q test. When substantial heterogeneity was present (I^2^ > 50% or Q-p < 0.10), a random-effects model was adopted to combine the results. Otherwise, a fixed-effects model was applied. To explore the sources of heterogeneity, we performed subgroup analyses based on geographic region (Asia, Europe, North America, and Africa), sample size (>200 vs ≤200), study design (retrospective vs prospective), number of centers (single-center vs multicenter), and radiotherapy dose. The conventional dose group was defined by a total radiotherapy dose of 45–50.4 Gy. The other/unreported dose group was defined by a dose lower than 45 Gy, higher than 50.4 Gy, short-course radiotherapy schedules, or an unreported radiotherapy dose. Leave-one-out sensitivity analysis was performed by sequentially excluding each study. Additional sensitivity analyses were performed by excluding the study with an unusually high yPNI prevalence and studies with potentially overlapping patient populations, and by restricting the analysis to studies that included both ypT and ypN stages as covariates in their multivariable models. Publication bias was appraised using Egger’s test and funnel plots. All meta-analyses were performed in Stata 15.1. A two-sided *P* < 0.05 was considered statistically significant.

## Results

### Literature retrieval and screening process

**Figure 1:**
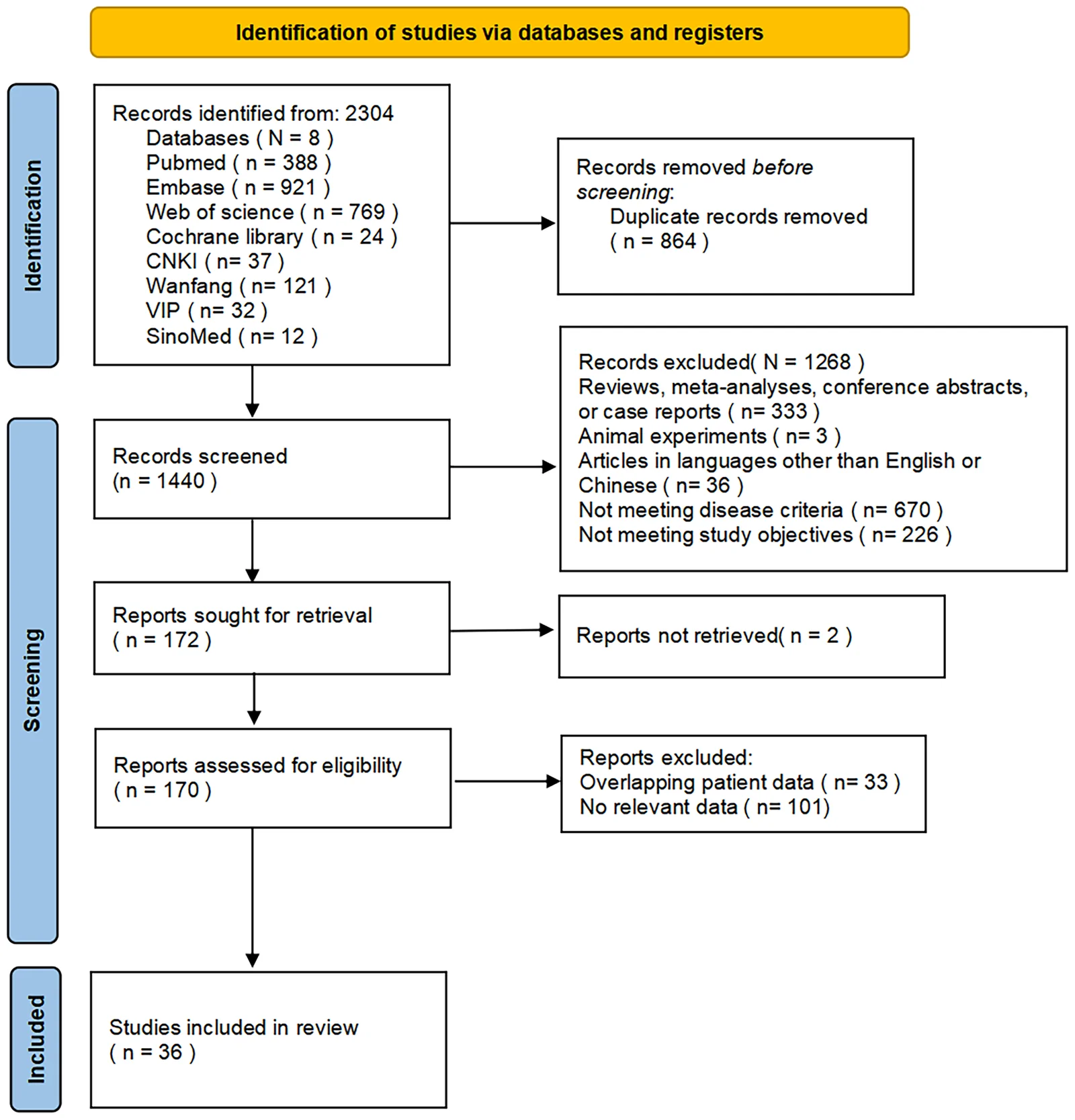
Flow chart of the study selection process.

A total of 2,304 records were initially identified from PubMed (n=388), Embase (n=921), Web of Science (n=769), Cochrane Library (n=24), CNKI (n =37), Wanfang Data (n =121), VIP (n =32), and SinoMed (n =12). After the removal of duplicate records (n=864), 1,440 records underwent title and abstract screening, and 1,268 were excluded. The remaining records were evaluated through full-text review in strict accordance with the predefined inclusion and exclusion criteria. Ultimately, 36 studies were included in this study (16–19, 22–53). The study screening was performed independently by two reviewers, and any divergences or uncertainties were settled by discussion. The screening process is detailed in **Figure 1**.

### Basic characteristics and quality assessment of included studies

This study included 36 cohort studies published between 2013 and 2025, comprising 18,721 individuals diagnosed with LARC. These studies were conducted across 9 countries (Republic of Korea, China, the United States, Turkey, France, Japan, Thailand, Portugal, and Egypt), predominantly in Asia. The age of the patients was 18 to 88 years. Males accounted for the highest proportion in each study (ranging from 52.0% to 73.3%). The incidence rate of yPNI varied across studies (from 2.9% to 66.8%), with a mean of 15.9%. The PNI definitions reported in the included studies are summarized in **Supplementary Appendix A3**. The detailed characteristics of the included studies are provided in **Table 1**.

**Table 1:** Baseline characteristics of included cohorts.

Study	Country	Institution or database	Enrollment years	Inclusion dates	Whether the cohort overlaps with another publication	Design	Center (s: single; m: multi)	Sample size	PNI (+)(n)(%)	PNI(%)	Age	Sex (man/woman)	Stage (AJCC)	Treatment	Data source	Outcome
Sano et al., 2025 (16)	Japan	Cancer Institute Hospital, Japanese Foundation for Cancer Research, Tokyo	2005–2014	NR	No	retrospective cohort	s	255	96	37.6		174/81	II-III	nCRT+radical operation	HR	OS,RFS
Mülküt et al., 2025 (16)	Turkey	Sancaktepe Şehit Prof. Dr. İlhan Varank Training and Research Hospital, University of Health Sciences, Istanbul, Turkey	2021–2024	NR	No	retrospective cohort	s	100	35	35	61.25 ± 13.20	52/48	II-III	nCRT+radical operation	HR	OS,DFS
Luo et al., 2025 (17)	China	Fudan University Shanghai Cancer Center, Shanghai, China	2010–2018	June 2010 to December 2018	No	retrospective cohort	s	671	107	15.9		465/206	II-III	nCRT+radical operation	HR	OS,DFS
Fan et al., 2025 (28)	China	Tianjin Medical University Cancer Institute & Hospital, National Clinical Research Center for Cancer, Tianjin, China	2019–2021	March 2019 to March 2021	No	retrospective cohort	s	143	21	14.7		77/66	II-III	nCRT+radical operation	HR	DFS
Pan et al., 2024 (19)	China	The First Affiliated Hospital of Fujian Medical University, Fuzhou	2013–2019	March 2013 to October 2019	No	retrospective cohort	s	513	25	4.9	18-80	329/184	II-III	nCRT+radical operation	HR	OS,DFS
Naovaset et al., 2024 (46)	Thailand	Faculty of Medicine Ramathibodi Hospital, Mahidol University, Thailand	2015–2019	NR	No	retrospective cohort	s	145	27	18.6	61.4 ± 10.9	93/52	II-III	nCRT+radical operation	HR	DFS, LRFS
Morais et al., 2024 (44)	Portugal	Unidade Local de Saúde de Matosinhos and Centro Hospitalar Universitário de São João, Porto, Portugal	2010–2022	January 2010 to December 2022	No	retrospective cohort	m	245	59	24.1	61 ± 12	151/94	II-III	nCRT+radical operation	HR	DFS
Hwang et al., 2024 (31)	Korea	Chonnam National University Hwasun Hospital, Hwasun, Republic of Korea	2015–2019	January 2015 to December 2019	No	retrospective cohort	s	108	47	43.5		79/29	III	nCRT+radical operation	HR	OS, RFS
Cai et al., 2024 (23)	China	The Sixth Affiliated Hospital, Sun Yat-sen University, China	2012–2019	March 2012 to September 2019	No	retrospective cohort	s	245	7	2.9		170/75	II-III	nCRT+radical operation	HR	DFS
Ulusoy et al., 2023 (53)	Turkey	Prof. Dr. Cemil Taşçıoğlu City Hospital, Istanbul, Türkiye	2016–2022	January 2016 to January 2022	No	retrospective cohort	s	154	47	30.5	61.1	105/49	II-III	nCRT+radical operation	HR	OS
Ryu et al., 2023 (48)	Korea	Asan Medical Center, University of Ulsan College of Medicine, Seoul, Republic of Korea	2007–2016	January 2007 to December 2016	Potential overlap	retrospective cohort	s	1240	210	16.9	57.4 ± 10.2	830/410	II-III	nCRT+radical operation	HR	OS, DFS
Rayan et al., 2023 (47)	Egypt	Faculty of Medicine, Assiut University, Assiut, Egypt	2015–2017	January 2015 to December 2017	No	retrospective cohort	s	100	18	18	43.1 ± 1.5	55/45	II-III	nCRT+radical operation	HR	LRFS
Lu et al., 2023 (42)	China	Peking University Third Hospital and Peking University People’s Hospital, Beijing, China	2012–2021	January 2012 to June 2021	No	retrospective cohort	m	172	13	7.6	63-74	113/59	II-III	nCRT+radical operation	HR	DFS
Liao et al., 2023 (39)	China	Surveillance, Epidemiology, and End Results(SEER)Program database	2010–2015	Diagnosed between 2010 and 2015	No	retrospective cohort	m	2957	371	12.5		1861/1096	II-III	nCRT+radical operation	HR	OS, CSS
Lim et al., 2022 (40)	Korea	Seoul National University Hospital, Seoul, Republic of Korea	NR	NR	No	retrospective cohort	s	165	15	9	29-86	116/49	II-III	nCRT+radical operation	HR	OS, DFS
Kim,S et al., 2022 (36)	Korea	Samsung Medical Center, Sungkyunkwan University School of Medicine, Seoul, Republic of Korea	2010–2015	January 2010 to December 2015	Potential overlap	retrospective cohort	s	602	402	66.8	28-83	408/194	II-III	nCRT+radical operation	HR	OS, DFS
DELİKGÖZ SOYKUT et al., 2022 (27)	Turkey	University of Health Sciences, Samsun Training and Research Hospital, Samsun, Türkiye	2012–2017	January 2012 to December 2017	No	retrospective cohort	s	55	22	40	30-83		II-III	nCRT+radical operation	HR	DFS, LRFS
Zheng et al., 2021 (52)	China	Fujian Medical University Union Hospital, Fuzhou, China	2011–2016	NR	No	retrospective cohort	s	785	30	3.8	55.9 ± 11.4	513/272	II-III	nCRT+radical operation	HR	OS, RFS
Naffouje et al., 2021 (45)	America	National Cancer Database(NCDB), United States	2010–2015	NR	No	retrospective cohort	m	3570	230	6.4	61.28 ± 11.48		II	nCRT+radical operation	HR	OS
Mei et al., 2020 (43)	China	National Cancer Center/Cancer Hospital, Chinese Academy of Medical Sciences and Peking Union Medical College, Beijing, China	2014–2017	November 2014 to August 2017	Potential overlap	retrospective cohort	s	231	54	23.4	51-66.5	160/71	II-III	nCRT+radical operation	HR	DFS
Liu et al., 2020 (41)	China	Cancer Hospital, Chinese Academy of Medical Sciences and Peking Union Medical College/National Cancer Center, Beijing, China	2010–2018	January 2010 to July 2018	Potential overlap	retrospective cohort	s	243	14	5.8	23-82	166/77	II-III	nCRT+radical operation	HR	OS, CSS
Sun et al., 2019 (50)	China	Zhejiang Cancer Hospital, Hangzhou, China	2002–2012	February 2002 to December 2012	No	retrospective cohort	s	181	44	24.3	23-76	129/52	II-III	nCRT+radical operation	HR	OS, DFS
Song et al., 2019 (49)	Korea	Six tertiary institutions in the Republic of Korea	2005–2014	NR	Potential overlap	retrospective cohort	m	1232	211	17.1	27-83	840/392	II-III	nCRT+radical operation	HR	LRFS, MeFS
Lee,J et al., 2019 (38)	Korea	Korean Radiation Oncology Group; eight tertiary institutions in the Republic of Korea	NR	NR	Potential overlap	retrospective cohort	m	1804	352	19.5		1238/566	II-III	nCRT+radical operation	HR	OS, RFS
Lee,H.G et al., 2019 (37)	Korea	Asan Medical Center, University of Ulsan College of Medicine, Seoul, Republic of Korea	2008–2011	January 2008 to November 2011	Potential overlap	retrospective cohort	s	389	76	19.5	25-79	254/135	II-III	nCRT+radical operation	HR	RFS
Kim,S.M et al., 2019 (36)	Korea	Kyungpook National University Medical Center, Daegu, Republic of Korea	2006–2011	January 2006 to December 2011	Potential overlap	retrospective cohort	s	146	34	23.3	29-85	102/44	II-III	nCRT+radical operation	HR	RFS, CSS
Kim,C.H et al., 2019 (34)	Korea	Chonnam National University Hwasun Hospital and Medical School, Gwangju, Republic of Korea	NR	NR	Potential overlap	retrospective cohort	s	446	126	28.3			II-III	nCRT+radical operation	HR	OS, DFS
Cho et al., 2019 (25)	Korea	Kyungpook National University Chilgok Hospital, Daegu, Republic of Korea	2006–2015	January 2006 to October 2015	Potential overlap	retrospective cohort	s	284	47	16.5	25-88	198/86	II-III	nCRT+radical operation	HR	OS, DFS
Kim et al., 2018 (35)	Korea	Ewha Womans University–affiliated institutional cohort, Seoul, Republic of Korea	2000–2015	NR	Potential overlap	retrospective cohort	s	60	12	20	26-82		II-III	nCRT+radical operation	HR	OS
Vychnevskaia et al., 2017 (51)	France	Ambroise Paré Hospital, Bicêtre Hospital, and Gustave Roussy Cancer Center, France	2007–2014	January 2007 to March 2014	No	prospective cohort	m	200	16	8	61.4 ± 11.4		II-III	nCRT+radical operation	HR	OS, DFS
Chablani et al., 2017 (24)	America	The Ohio State University, Columbus, Ohio, USA	2004–2011	NR	No	retrospective cohort	s	87	14	16	22-81		II-III	nCRT+radical operation	HR	DFS, MeFS
Huh et al., 2016 (30)	Korea	Samsung Medical Center, Sungkyunkwan University School of Medicine, Seoul, Republic of Korea	NR	NR	Potential overlap	retrospective cohort	s	209	17	8.1	27-81	136/73	II-III	nCRT+radical operation	HR	OS
Kim et al., 2015 (33)	Korea	Chonnam National University Hwasun Hospital and Medical School, Gwangju, Republic of Korea	2005–2012	February 2005 to December 2012	Potential overlap	retrospective cohort	s	419	108	25.8	62.12 ± 0.93	307/112	II-III	nCRT+radical operation	HR	LRFS, MeFS
Huh et al., 2015 (29)	Korea	Samsung Medical Center, Sungkyunkwan University School of Medicine, Seoul, Republic of Korea	2007–2011	January 2007 to March 2011	Potential overlap	retrospective cohort	s	210	17	8.1	31-79	151/59	II-III	nCRT+radical operation	HR	LRFS
Chung et al., 2013 (26)	Korea	Uijeongbu St. Mary’s Hospital, The Catholic University of Korea, Seoul/Uijeongbu, Republic of Korea	1996–2010	July 1996 to June 2010	No	retrospective cohort	s	104	22	21.2	28-85	71/33	II-III	nCRT+radical operation	HR	OS, DFS, MeFS
Agarwal et al., 2013 (22)	America	The University of Texas MD Anderson Cancer Center institutional colorectal cancer database and tumor registry, Houston, USA	2000–2008	NR	No	retrospective cohort	s	251	26	10.4		157/94	II-III	nCRT+radical operation	HR	RFS

The quality scores of the included studies ranged from 8 to 9, indicating high quality (**Table 2**).

**Table 2:** Quality assessment of included studies.

	Selection				Comparability	Outcome			Quality scores
	Representativeness of the exposed cohort	Selection of the nonexposed cohort	Ascertainment of exposure	Demonstration that outcome of interest was not present at start of study	Comparability of cohorts on the basis of the design or analysis	Assessment of outcome	Was follow-up long enough for outcomes to occur	Adequacy of follow up of cohorts	
Sano et al.2025 (18)	1	1	1	1	2	1	1	1	9
Mülküt et al.2025 (16)	1	1	1	1	2	1	1	0	8
Luo et al.2025 (17)	1	1	1	1	2	1	1	1	9
Fan et al.2025 (28)	1	1	1	1	2	1	1	0	8
Pan et al.2024 (19)	1	1	1	1	2	1	1	1	9
Naovaset et al.2024 (46)	1	1	1	1	2	1	1	0	8
Morais et al.2024 (44)	1	1	1	1	2	1	1	1	9
Hwang et al.2024 (31)	1	1	1	1	2	1	1	1	9
Cai et al.2024 (23)	1	1	1	1	2	1	1	1	9
Ulusoy et al.2023 (53)	1	1	1	1	2	1	1	1	9
Ryu et al.2023 (48)	1	1	1	1	2	1	1	1	9
Rayan et al.2023 (47)	1	1	1	1	2	1	1	1	9
Lu et al.2023 (42)	1	1	1	1	2	1	1	1	9
Liao et al.2023 (39)	1	1	1	1	2	1	1	0	8
Lim et al.2022 (40)	1	1	1	1	2	1	1	1	9
Kim,S et al.2022 (36)	1	1	1	1	2	1	1	0	8
DELİKGÖZ SOYKUT et al.2022 (27)	1	1	1	1	2	1	1	1	9
Zheng et al.2021 (52)	1	1	1	1	2	1	1	1	9
Naffouje et al.2021 (45)	1	1	1	1	2	1	1	1	9
Mei et al.2020 (43)	1	1	1	1	2	1	1	1	9
Liu et al.2020 (41)	1	1	1	1	2	1	1	1	9
Sun et al.2019 (50)	1	1	1	1	2	1	1	0	8
Song et al., 2019 (49)	1	1	1	1	2	1	1	1	9
Lee,J et al.2019 (38)	1	1	1	1	2	1	1	1	9
Lee,H.G et al.2019 (37)	1	1	1	1	2	1	1	1	9
Kim,S.M et al.2019 (36)	1	1	1	1	2	1	1	1	9
Kim,C.H et al.2019 (34)	1	1	1	1	2	1	1	1	9
Cho et al.2019 (25)	1	1	1	1	2	1	1	1	9
Kim et al.2018 (35)	1	1	1	1	2	1	1	0	8
Vychnevskaia et al.2017 (51)	1	1	1	1	2	1	1	1	9
Chablani et al.2017 (24)	1	1	1	1	2	1	1	0	8
Huh et al.2016 (30)	1	1	1	1	2	1	1	1	9
Kim et al.2015 (33)	1	1	1	1	2	1	1	1	9
Huh et al.2015 (29)	1	1	1	1	2	1	1	1	9
Chung et al.2013 (26)	1	1	1	1	2	1	1	0	8
Agarwal et al.2013 (22)	1	1	1	1	2	1	1	1	9

## Results of meta-analysis

### OS

**Figure 2:**
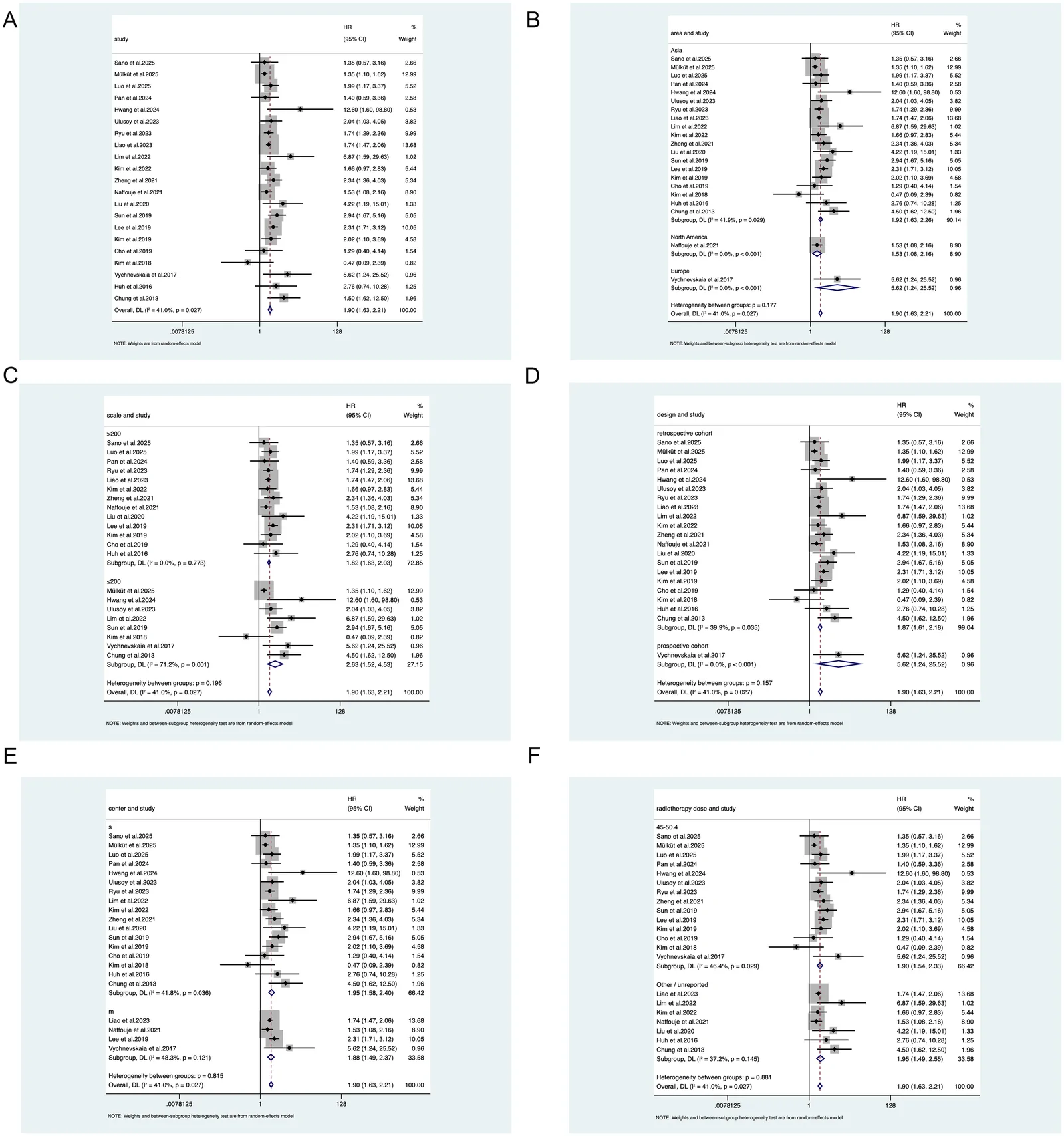
Meta-analysis of the effect of yPNI on OS in patients. **(A)** Forest plot of the overall meta-analysis; **(B)** Forest plot of the subgroup analysis stratified by geographic region; **(C)** Forest plot of the subgroup analysis stratified by sample size; **(D)** Forest plot of the subgroup analysis stratified by study design; **(E)** Forest plot of the subgroup analysis stratified by number of centers; **(F)** Forest plot of the subgroup analysis stratified by radiotherapy dose. OS, overall survival; yPNI, post-nCRT perineural invasion.

A total of 21 studies involving 14,651 individuals evaluated this outcome (**Figure 2**). Moderate heterogeneity was detected across studies (I^2^ = 41.0%, *P* = 0.027). Thus, a random-effects model was used. The pooled OS results showed that the yPNI-positive group was markedly associated with adverse OS (HR = 1.90, 95% CI: 1.63-2.21, *P* < 0.001). Due to the moderate heterogeneity, subgroup analyses were performed according to study region, study size, study design, number of centers, and radiotherapy dose.

By geographic region, the Asia subgroup (19 studies) had an HR of 1.92 (1.63-2.26) with significant heterogeneity (I^2^ = 41.9%, *P* = 0.029); Europe and North America: only one study. No significant difference was detected across geographic subgroups (*P* for subgroup difference = 0.177). According to sample size, large (>200, 13 studies): HR 1.82 (1.63-2.03), no heterogeneity (I^2^ = 0%, *P* = 0.773); small (≤200, 8 studies): HR 2.63 (1.52-4.53), significant heterogeneity (I^2^ = 71.2%, *P* = 0.001). There was no significant difference between subgroups (*P* for subgroup difference = 0.196). Based on the number of centers, single-center (17 studies): HR 1.95 (1.58-2.40), significant heterogeneity (I^2^ = 41.8%, *P* = 0.036); multi-center (4 studies): HR 1.88 (1.49-2.37), no heterogeneity (I^2^ = 48.3%, *P* = 0.121). There was no significant difference between subgroups (*P* for subgroup difference = 0.815). By study design, retrospective (20 studies): HR 1.87 (1.61-2.18), significant heterogeneity (I^2^ = 39.9%, *P* = 0.035); prospective: only one study. There was no significant difference between subgroups (*P* for subgroup difference = 0.157). By radiotherapy dose, conventional (14 studies): HR 1.90 (1.54-2.33), significant heterogeneity (I^2^ = 46.4%, *P* = 0.029); other/unreported (7 studies): HR 1.95 (1.49–2.55), no heterogeneity (I^2^ = 37.2%, *P* = 0.145). There was no significant difference between subgroups (*P* for subgroup difference = 0.881). None of the above subgroup analyses clearly identified the source of heterogeneity in OS.

### RFS

**Figure 3:**
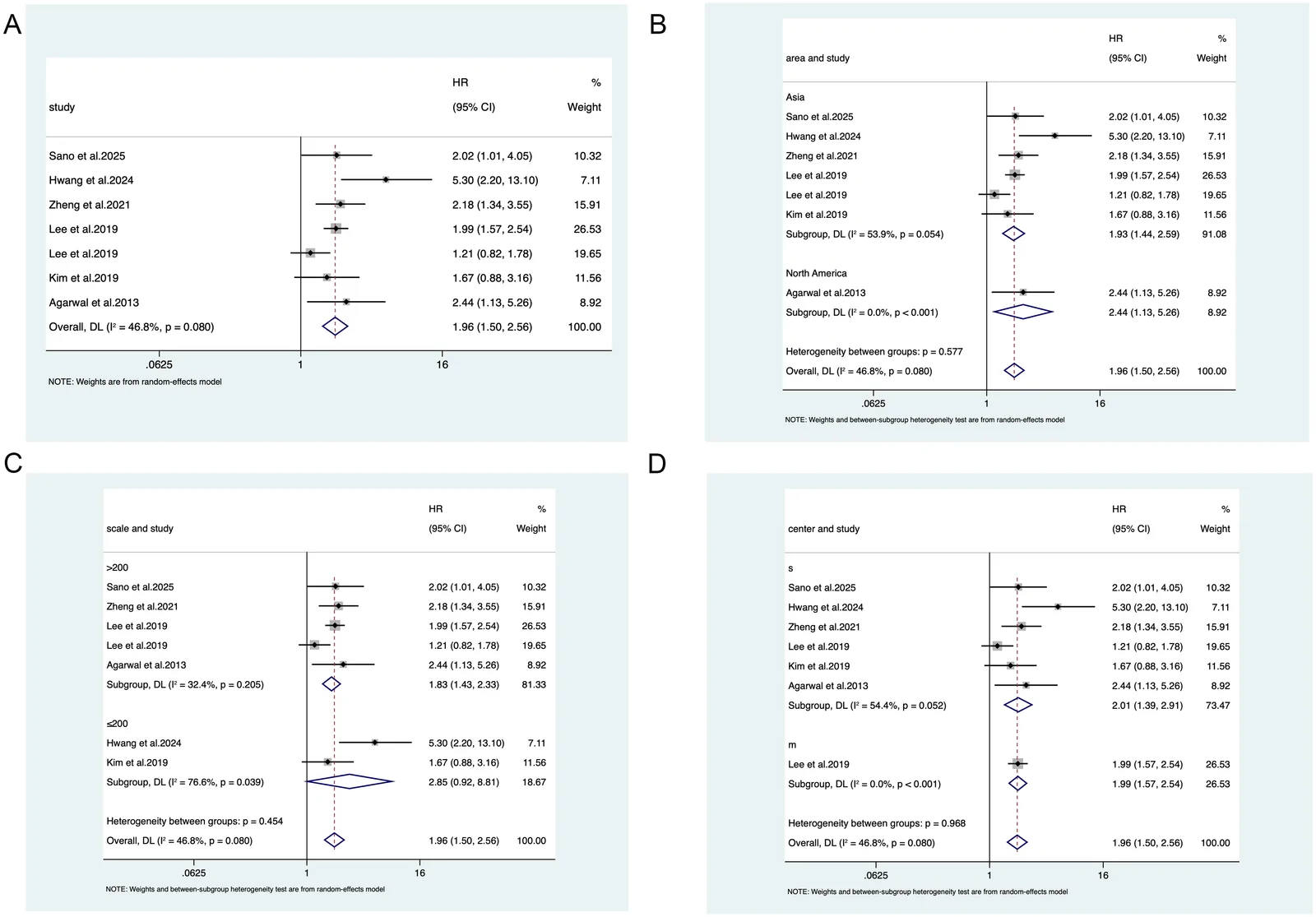
Meta-analysis of the association between yPNI and RFS. **(A)** Forest plot of the overall meta-analysis; **(B)** Forest plot of the subgroup analysis stratified by geographic region; **(C)** Forest plot of the subgroup analysis stratified by sample size; **(D)** Forest plot of the subgroup analysis stratified by number of centers. RFS, recurrence-free survival; yPNI, post-nCRT perineural invasion.

A total of 7 studies comprising 3,738 individuals analyzed this outcome (**Figure 3**). Moderate heterogeneity was identified across studies (I^2^ = 46.8%, *P* = 0.080). Hence, a random-effects model was adopted. The pooled analysis showed that yPNI positivity was significantly associated with poorer RFS (HR = 1.96, 95% CI: 1.50-2.56, *P* < 0.001). Subgroup analyses were carried out in accordance with study region, study size, and number of centers.

By geographic region, the Asia subgroup (6 studies) had a pooled HR of 1.93 (95% CI: 1.44-2.59), with moderate heterogeneity (I^2^ = 53.9%, *P* = 0.054), whereas the only study from North America reported an HR of 2.44 (95% CI: 1.13-5.26). There was no significant difference between geographic subgroups (*P* for subgroup difference = 0.577). According to the number of centers, single-center studies (6 studies) showed a pooled HR of 2.01 (95% CI: 1.39-2.91), with significant heterogeneity (I^2^ = 54.4%, P = 0.052), while the only multicenter study reported an HR of 1.99 (95% CI: 1.57-2.54). No significant difference was observed between the subgroups (*P* for subgroup difference = 0.968). By sample size, studies including more than 200 patients (5 studies) had a pooled HR of 1.83 (95% CI: 1.43-2.33), with low heterogeneity (I^2^ = 32.4%, *P* = 0.205), whereas studies including 200 patients or fewer (2 studies) had a pooled HR of 2.85 (95% CI: 0.92-8.81), with substantial heterogeneity (I^2^ = 76.6%, *P* = 0.039). There was no significant difference between the sample-size subgroups (*P* for subgroup difference = 0.454). All included studies were retrospective cohort studies and fell into the conventional-dose subgroup. Therefore, subgroup comparisons according to study design or radiotherapy dose could not be performed. None of the evaluable subgroup factors clearly explained the between-study heterogeneity in RFS.

### DFS

**Figure 4:**
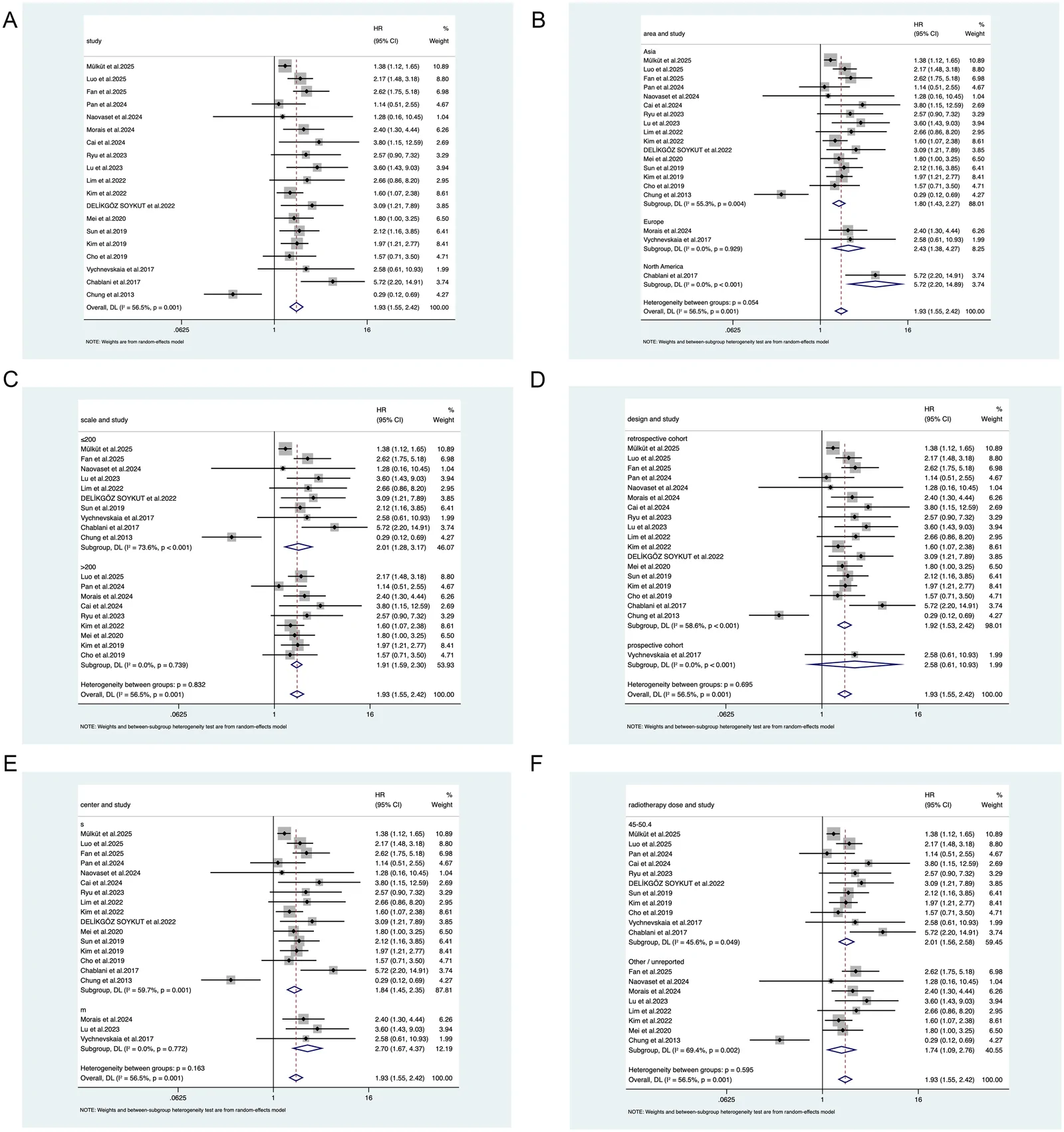
Meta-analysis of the association between yPNI and DFS. **(A)** Forest plot of the overall meta-analysis; **(B)** Forest plot of the subgroup analysis stratified by geographic region; **(C)** Forest plot of the subgroup analysis stratified by sample size; **(D)** Forest plot of the subgroup analysis stratified by study design; **(E)** Forest plot of the subgroup analysis stratified by number of centers; **(F)** Forest plot of the subgroup analysis stratified by radiotherapy dose. DFS, disease-free survival; yPNI, post-nCRT perineural invasion.

A total of 19 studies comprising 5,829 patients evaluated DFS (**Figure 4**). Marked heterogeneity was identified across studies (I^2^ = 56.5%, *P* = 0.001). Hence, a random-effects model was adopted. The pooled analysis revealed that yPNI positivity was significantly associated with poorer DFS in patients with rectal cancer undergoing nCRT (HR = 1.93, 95% CI: 1.55-2.42, *P* < 0.001). Due to the marked heterogeneity, subgroup analyses were carried out in accordance with study region, study size, study design, number of centers, and radiotherapy dose.

By geographic region, the Asia subgroup (16 studies) had an HR of 1.80 (95% CI: 1.43-2.27) with significant heterogeneity (I^2^ = 55.3%, *P* = 0.004); Europe (2 studies): HR 2.43 (95% CI: 1.38-4.27), no heterogeneity (I^2^ = 0%, *P* = 0.929); the only study from North America reported an HR of 5.72 (95% CI: 2.20-14.89). There was no significant difference between subgroups (*P* for subgroup difference = 0.054). According to the number of centers, single-center (16 studies): HR 1.84 (95% CI: 1.45-2.35), significant heterogeneity (I^2^ = 59.7%, *P* = 0.001); multi-center (3 studies): HR 2.70 (95% CI: 1.67-4.37), no heterogeneity (I^2^ = 0%, *P* = 0.772). There was no significant difference between subgroups (*P* for subgroup difference = 0.163). Based on study design, retrospective (18 studies): HR 1.92 (95% CI: 1.53-2.42), significant heterogeneity (I^2^ = 58.6%, *P* < 0.001); prospective: only one study. There was no significant difference between subgroups (*P* for subgroup difference = 0.695). By sample size, small (≤200, 10 studies): HR 2.01 (95% CI: 1.28-3.17), with substantial heterogeneity (I^2^ = 73.6%, *P* < 0.001); large (>200, 9 studies): 1.91 (95% CI: 1.59-2.30), with no detected heterogeneity (I^2^ = 0%, *P* = 0.739). There was no significant difference between subgroups (*P* for subgroup difference = 0.832). By radiotherapy dose, conventional (45–50.4 Gy, 11 studies): HR 2.01 (95% CI: 1.56-2.58), moderate heterogeneity (I^2^ = 45.6%, *P* = 0.049); other/unreported (8 studies): HR 1.74 (95% CI: 1.09-2.76), with substantial heterogeneity (I^2^ = 69.4%, *P* = 0.002). There was no significant difference between subgroups (*P* for subgroup difference = 0.595).

Overall, none of the prespecified subgroup variables clearly explained the between-study heterogeneity in DFS. However, we noted that in one prospective study, no substantial association was identified, which might be attributed to the small sample size.

### CSS

**Figure 5:**
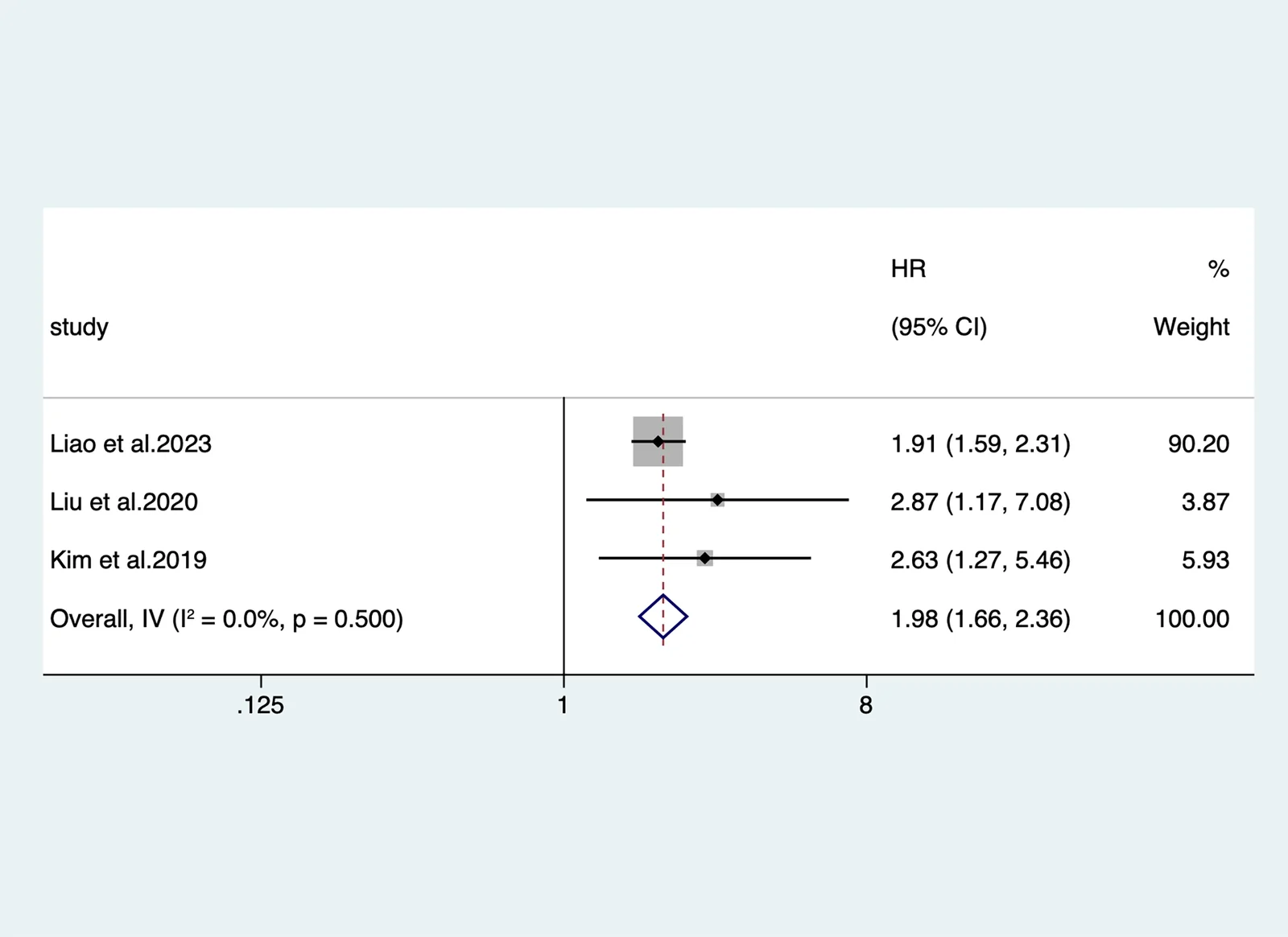
Meta-analysis of the association between yPNI and CSS. Note: CSS, cancer-specific survival; yPNI, post-nCRT perineural invasion.

A total of 3 studies with 3,346 individuals examined this outcome (**Figure 5**). No substantial heterogeneity was found across studies (I^2^ = 0.0%, *P* = 0.500). Thus, a fixed-effects model was adopted. The pooled CSS results demonstrated that the yPNI-positive group was associated with adverse CSS (HR = 1.98, 95% CI: 1.66-2.36, *P* < 0.001). Notably, in this analysis, a single study by Liao et al. (39) accounted for 90.2% of the weight (39), and the pooled effect mainly reflected the findings of that study. Therefore, this result was considered hypothesis-generating and a cautious interpretation was required.

### LRFS

**Figure 6:**
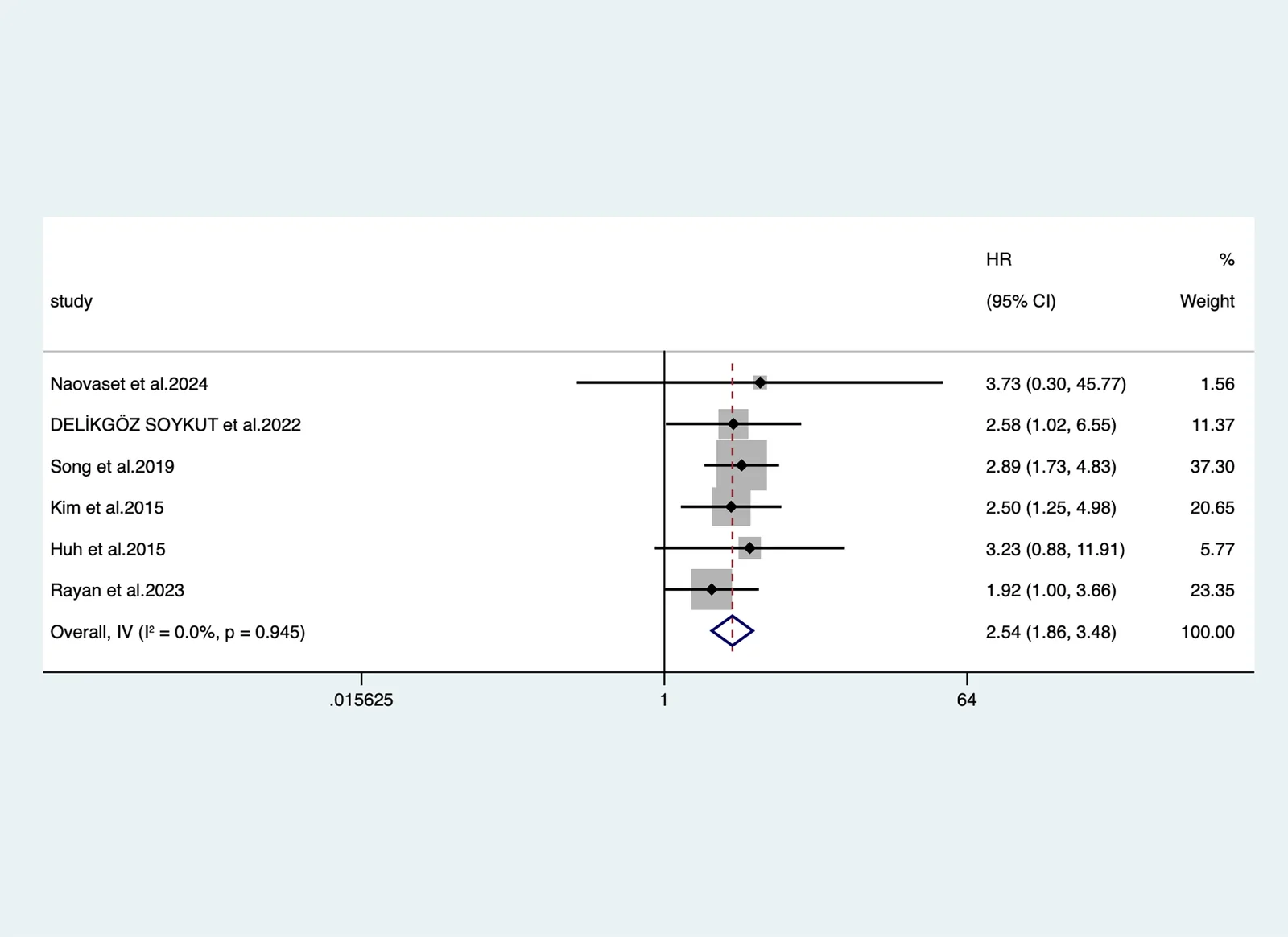
Meta-analysis of the association between yPNI and LRFS. Note: LRFS, local recurrence-free survival; yPNI, post-nCRT perineural invasion.

In total, 6 studies involving 2,161 participants investigated this outcome (**Figure 6**). No marked heterogeneity was observed across studies (I^2^ = 0%, *P* = 0.945). Thus, a fixed-effects model was used. The pooled LRFS results showed that the yPNI-positive group was significantly associated with adverse LRFS (HR = 2.54, 95% CI: 1.86-3.48, *P* < 0.001).

### MeFS

**Figure 7:**
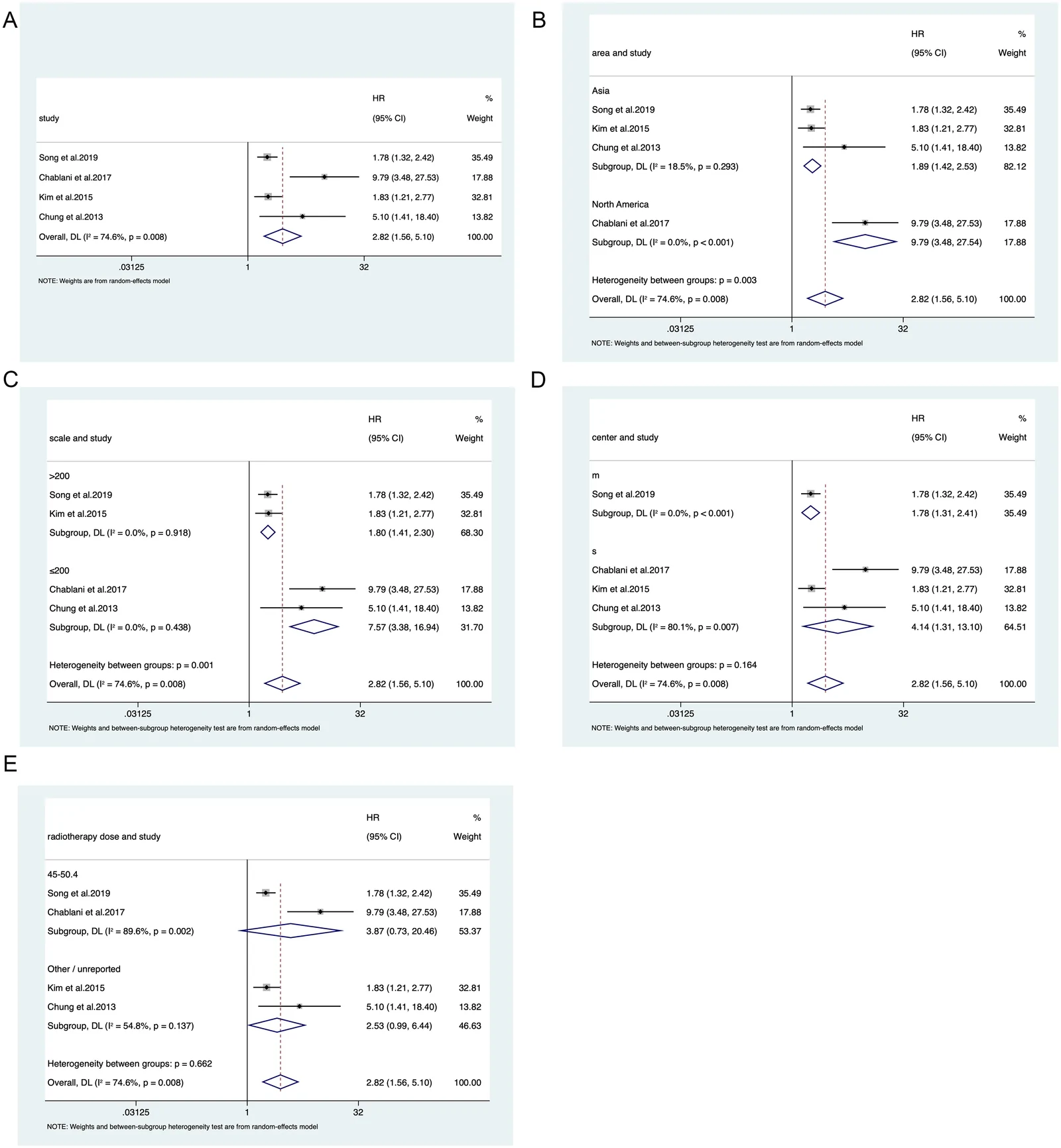
Meta-analysis of the association between yPNI and MeFS. **(A)** Forest plot of the overall meta-analysis; **(B)** Forest plot of the subgroup analysis stratified by geographic region; **(C)** Forest plot of the subgroup analysis stratified by sample size; **(D)** Forest plot of the subgroup analysis stratified by number of centers; **(E)** Forest plot of the subgroup analysis stratified by radiotherapy dose. Note: MeFS, metastasis-free survival; yPNI, post-nCRT perineural invasion.

A total of 4 studies involving 1,842 patients examined this outcome (**Figure 7**). Marked heterogeneity was found across studies (I^2^ = 74.6%, *P* = 0.008). Therefore, a random-effects model was adopted. The pooled MeFS results demonstrated that the yPNI-positive group was significantly associated with distant metastasis (HR = 2.82, 95% CI: 1.56-5.10, *P* < 0.001). Due to substantial heterogeneity, further subgroup analyses were carried out according to study region, study size, number of centers, and radiotherapy dose.

By geographic region, the pooled HR was 1.89 (95% CI: 1.42-2.53; I^2^ = 18.5%, *P* = 0.293) across three Asian studies, whereas the only study from North America reported an HR of 9.79 (95% CI: 3.48-27.53). A significant difference was observed between the geographic subgroups (*P* for subgroup difference = 0.003). According to the number of centers, the single multicenter study reported an HR of 1.78 (95% CI: 1.32-2.42), whereas the three single-center studies yielded a pooled HR of 4.14 (95% CI: 1.31-13.10; I^2^ = 80.1%, *P* = 0.007). There was no significant difference between subgroups (*P* for subgroup difference = 0.164). By sample size, large (>200, 2 studies): HR 1.80 (95% CI: 1.41-2.30), no heterogeneity (I^2^ = 0%, *P* = 0.918); small (≤200, 2 studies): HR 7.57 (3.38–16.94), no heterogeneity (I^2^ = 0%, *P* = 0.438). There was a significant difference between subgroups (*P* for subgroup difference = 0.001). By radiotherapy dose, conventional (45–50.4 Gy, 2 studies): HR 3.87 (95% CI: 0.73-20.46), significant heterogeneity (I^2^ = 89.6%, *P* = 0.002); the other/unreported-dose subgroup (2 studies) had a pooled HR of 2.53 (95% CI: 0.99-6.44; I^2^ = 54.8%, *P* = 0.137). No significant difference was observed between the dose subgroups (*P* for subgroup difference = 0.662). Because all studies were retrospective, subgroup analysis by study design was not performed.

Significant subgroup differences were observed for geographic region and sample size. However, these findings were based on a limited number of studies and should be interpreted cautiously.

## Sensitivity analysis

**Figure 8:**
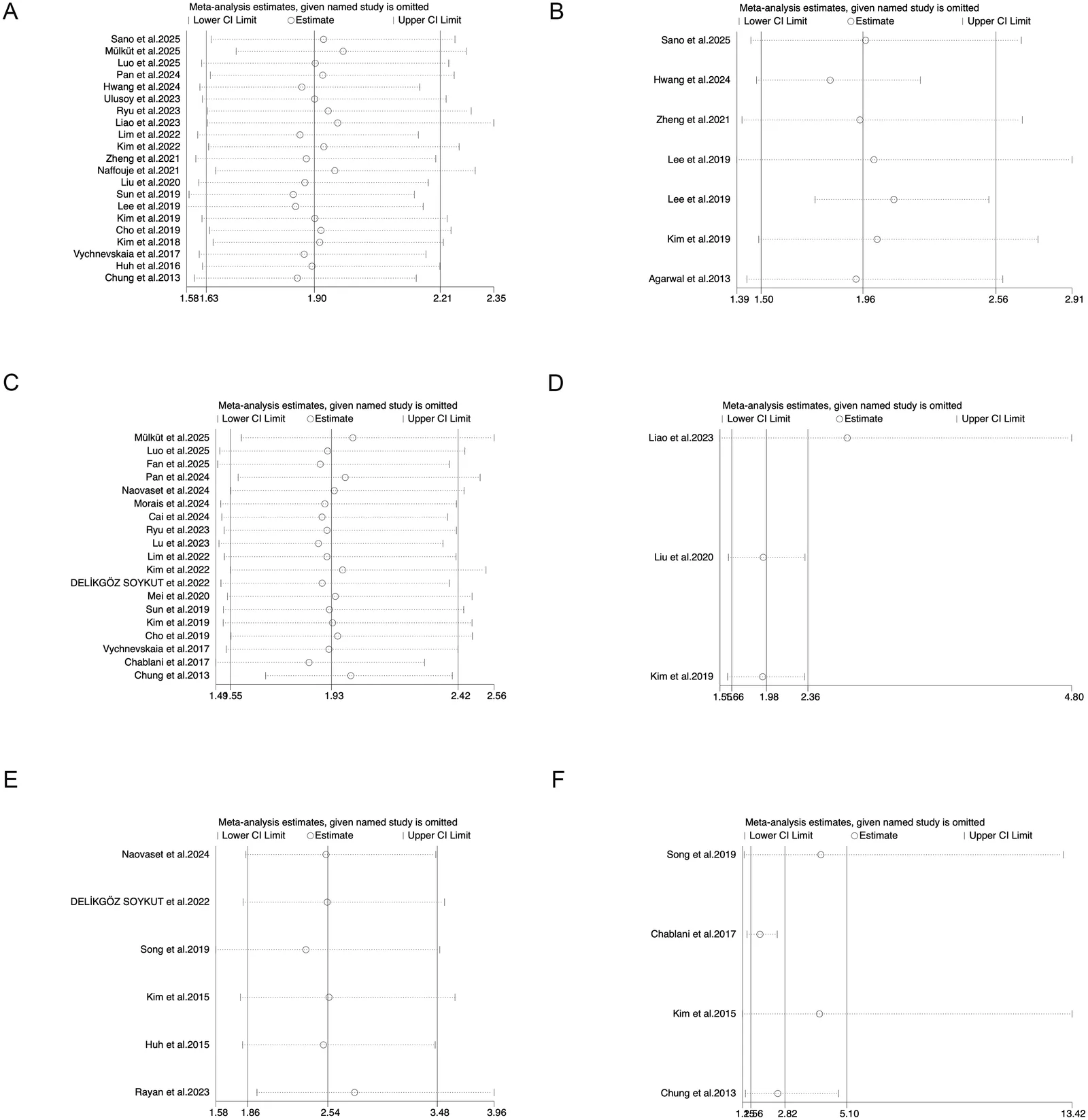
Leave-one-out sensitivity analyses for **(A)** OS, **(B)** RFS, **(C)**DFS, **(D)** CSS, **(E)** LRFS and **(F)** MeFS. Note: OS, overall survival; RFS, recurrence-free survival; DFS, disease-free survival; CSS, cancer-specific survival; LRFS, local recurrence-free survival; MeFS, metastasis-free survival.

Leave-one-out sensitivity analyses were performed for OS, RFS, DFS, CSS, LRFS, and MeFS by sequentially omitting individual studies to appraise the robustness of the conclusions. The results indicated that the pooled HRs and their 95% CIs remained largely unchanged, suggesting robust findings (**Figure 8**).

In an additional sensitivity analysis, studies reporting RFS or DFS were pooled to generate a broader recurrence-related outcome. The pooled analysis showed that yPNI positivity was associated with poorer recurrence-related survival (HR = 1.94, 95% CI: 1.64-2.30; I^2^ = 53.2%, *P* < 0.001), consistent with the separate RFS and DFS analyses (**Supplementary Appendix A4A**).

Given that the yPNI detection rate reported by Kim S et al. (32) (66.8%) was much higher than the rates reported in the other studies (2.9%–43.5%) (32), we recalculated the pooled estimates after excluding that study to assess the impact of this outlier on the pooled effect estimates. The results showed no substantial impact on the main conclusions for OS (HR = 1.92, 95% CI: 1.64-2.26, *P* = 0.019) and DFS (HR = 1.98, 95% CI: 1.55-2.53, *P* < 0.001) (**Supplementary Appendix A4B**).

Further sensitivity analyses addressed potential cohort overlap and differences in covariate adjustment. After excluding studies with potentially overlapping populations, yPNI positivity remained significantly associated with poorer OS (HR = 1.88, 95% CI: 1.61-2.18; I^2^ = 41.2%, *P* = 0.039) and RFS (HR = 1.83, 95% CI: 1.54-2.17; I^2^ = 16.7%, *P* = 0.306) (**Supplementary Appendix A4C**).

When the analyses were restricted to studies that included both ypT and ypN stage as covariates in their multivariable models, the associations remained significant for OS (HR = 1.90, 95% CI: 1.70-2.13; I^2^ = 0%, *P* = 0.491), RFS (HR = 1.99, 95% CI: 1.64-2.42; I^2^ = 0%, *P* = 0.935), DFS (HR = 2.04, 95% CI: 1.73-2.40; I^2^ = 0%, *P* = 0.519), LRFS (HR = 2.47, 95% CI: 1.65-3.69; I^2^ = 0%, *P* = 0.330), and MeFS (HR = 1.80, 95% CI: 1.41-2.30; I^2^ = 0%, *P* = 0.918) (**Supplementary Appendix A4D**). Collectively, these analyses supported the stability of the associations between yPNI positivity and poorer survival outcomes.

### Publication bias

**Figure 9:**
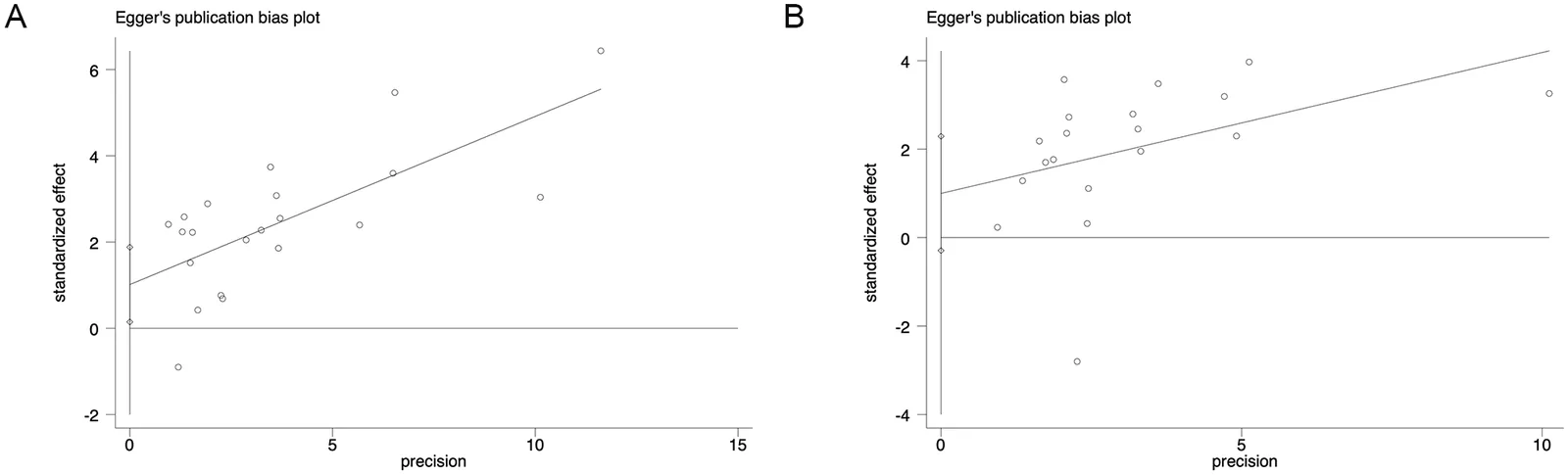
Publication bias assessment using **(A)** OS and **(B)** DFS. Note: OS, overall survival; DFS, disease-free survival.

Publication bias was assessed for outcomes including more than 10 studies, namely OS (**Figure 9A**) and DFS (**Figure 9B**). For DFS, Egger’s regression test did not indicate significant funnel plot asymmetry (*P* = 0.121). For OS, Egger’s test suggested significant funnel plot asymmetry (*P* = 0.024), indicating potential small-study effects or publication bias. An exploratory trim-and-fill analysis subsequently imputed four potentially missing studies. After imputation, the pooled random-effects estimate was attenuated from an HR of 1.90 (95% CI: 1.63-2.21) to 1.81 (95% CI: 1.53-2.14), while the direction and statistical significance of the association remained unchanged. These findings suggest that potential missing studies may have led to a modest overestimation of the association, but did not alter the qualitative conclusion regarding the association between yPNI positivity and poorer OS (**Supplementary Appendix A5**).

## Discussion

Currently, post-nCRT pathological evaluation primarily focuses on lymph node status and tumor regression grade (TRG). However, systematic evidence regarding the prognostic utility of yPNI is still lacking (54–56). To our knowledge, this is the first meta-analysis to investigate the prognostic significance of yPNI in patients with rectal cancer. Pooled analysis of 36 studies showed that yPNI was associated with adverse survival outcomes, including poorer OS, RFS, DFS, CSS, LRFS and MeFS. These findings support a prognostic association but do not determine whether yPNI improves prediction beyond established clinicopathological variables. The covariates included in the multivariable models varied across studies. Because yPNI is associated with poor tumor regression and advanced post-treatment stage, residual confounding by tumor burden cannot be excluded. Conversely, adjustment for downstream pathological factors may lead to overadjustment and attenuate the observed association. Therefore, the pooled adjusted HRs should not be interpreted as establishing yPNI as an independent prognostic predictor. Moreover, these results remained robust across subgroup analyses by region, sample size, study design and radiotherapy dose.

Previous literature has well established that, in CRC not treated with nCRT and in most solid tumors, PNI is widely recognized as a mark of aggressive tumor biology and is highly related to elevated tumor recurrence and poorer survival outcomes (13, 57, 58). A meta-analysis by Murugesan et al. involving 9,030 patients with rectal cancer has confirmed that PNI substantially increases the risk of mortality and recurrence (59). Based on these findings, the present study further demonstrated that yPNI remained significantly associated with poor prognosis in rectal cancer patients who had undergone nCRT. This finding suggests that the association between yPNI and adverse survival outcomes may persist after nCRT. Although no marked association between yPNI and prognosis is identified in a few studies, this inconsistency may be attributable to the relatively small sample sizes of individual studies (resulting in insufficient statistical power) or to inconsistencies in the criteria for yPNI assessment (29, 32, 46). Sensitivity analyses further confirmed the consistency of the overall results.

The detection rates of yPNI varied considerably across the included studies. This result may reflect heterogeneity in pathological assessment techniques and differences in yPNI definitions across studies (60). Conventional H&E staining may fail to identify some foci of yPNI when neural structures are obscured by perineural inflammation or mucin. S100 immunohistochemistry can highlight nerves and may facilitate PNI detection; however, its utility in post-nCRT rectal cancer specimens is extrapolated from other settings and requires further validation (61). Moreover, yPNI often presents as a focal and minimal lesion, which can be easily missed on a single section. Thus, review of multiple sections is required. Regarding the definition of yPNI, only some of the 36 studies provided clear diagnostic criteria, identifying two main definitions: tumor cells invading any layer of the nerve sheath, or involvement of at least one-third of the nerve circumference. Most studies did not specify the criteria. Of note, Kim S et al. (32) reported a yPNI detection rate as high as 66.8%, far exceeding that of other studies. That study did not provide a clear definition of PNI and may have adopted broader criteria (32). Sensitivity analysis showed that after excluding that study, the pooled effect size changed minimally. This indicated that this extreme value did not affect the main conclusions. Future studies should establish standardized definitions for yPNI after nCRT to improve comparability.

The pooled prevalence of yPNI in the current study was 15.9%, lower than the 26.5% reported for patients who did not receive nCRT (60). Treatment-induced necrosis, fibrosis, and mucinous degeneration after nCRT may obscure small perineural lesions and reduce the detection of yPNI. We hypothesize that yPNI persisting after nCRT may represent residual tumor with aggressive biological features, although this hypothesis remains to be validated.

The biological basis of this observation may involve tumor–nerve interactions within the neural microenvironment. In rectal cancer, PNI involves neural signaling and stromal crosstalk that contribute to tumor invasion and perineural spread (62–68).

TRG is a standard indicator for assessing pathological response after nCRT (69, 70). However, the direction of the grading scale varies across systems (71). Therefore, higher or lower TRG values should be interpreted according to the specific system used. For example, in the AJCC/CAP system, lower TRG values indicate a better pathological response (70). Emerging evidence suggests that these neural interactions may influence tumor proliferation, migration, and survival signaling. Nevertheless, whether they contribute to the persistence and prognostic significance of yPNI after nCRT remains unclear (72). Multiple studies included in this study also showed that yPNI positivity was more frequent in patients with poorer tumor regression. Agarwal et al. (22) demonstrated that the yPNI positivity rate increases from 0% in the complete pathologic response group to 18.2% in the minor pathologic response group (P<0.01) (22). Kim et al. (32) reported that the yPNI positivity rate is only 8.8% in the good response group, but as high as 91.2% in the poor response group (P<0.001) (73). Therefore, yPNI positivity is closely associated with poor tumor regression, further supporting the value of yPNI as a poor prognostic marker after nCRT.

However, the exact molecular mechanisms underlying yPNI-specific radioresistance remain incompletely understood and warrant further investigation.

Subgroup analyses for OS, RFS, DFS, and MeFS generally showed directionally consistent associations across subgroups, although the magnitude of the effect estimates varied. No definite source of heterogeneity was identified for OS, RFS, or DFS. For MeFS, the heterogeneity may be linked to the geographic region and sample size. Notably, the single prospective study did not demonstrate a significant association between yPNI and DFS. This finding is highly likely to be affected by insufficient statistical power due to its small sample size. Thus, further validation should be performed through more prospective cohort studies.

Several limitations of this study should be noted. First, most included studies were retrospective in design, which may have introduced selection bias, inadequate control for confounders, and information bias. Only one included study used a prospective design, limiting the strength of the available prospective evidence. Second, most included studies were from East Asia. Thus, extrapolation of our findings to Western populations should be made with caution. There are substantial differences in treatment strategies between Eastern and Western countries. The Japanese JSCCR guidelines routinely recommend lateral lymph node dissection for T3-4 rectal cancer, whereas the ESMO/NCCN guidelines rely more on nCRT. In Eastern countries, conventional long-course chemoradiotherapy is commonly used, while Western countries have more extensively explored the total neoadjuvant therapy (TNT) model combining short-course radiotherapy with chemotherapy. Differences also exist between Eastern and Western countries in terms of chemotherapy regimens, including drug selection, timing, and cycles (2, 4, 74). Moreover, there are differences in genetic background (e.g., KRAS/BRAF mutation frequencies) (75) and pathological criteria for PNI assessment between Eastern and Western patients, which may affect the detection rate and prognostic value of yPNI. Third, although we used the NOS for quality assessment in this meta-analysis, its application may involve subjective judgments (21). Fourth, regarding publication bias, for OS, four studies were imputed by the trim-and-fill method in the lower-left quadrant of the funnel plot, with effect sizes close to the line of no effect or in the opposite direction, suggesting possible under-publication of small-sample negative results. The asymmetry of the funnel plot for OS (**Figure 9A**) may be partly attributable to clinical heterogeneity, including differences in chemotherapy regimens and PNI definitions. For outcomes with fewer than 10 included studies (RFS, CSS, LRFS and MeFS), formal publication bias assessment was not feasible. Therefore, the potential influence of publication bias cannot be excluded. Notably, only four studies were included in the MeFS analysis, with substantial heterogeneity. Thus, the relevant conclusions should be interpreted cautiously. Future systematic reviews should expand the search scope to include grey literature and additional regional databases, such as Korean databases, to further reduce potential bias. Fifth, the definition and pathological assessment of yPNI were not fully standardized across studies, potentially resulting in misclassification bias. Finally, all analyses were based on post-nCRT surgical specimens. Therefore, our findings apply only to LARC patients who underwent radical surgery and cannot be generalized to those managed with a watch-and-wait strategy (2).

## Conclusion

The findings support an association between yPNI and adverse survival outcomes in rectal cancer patients treated with nCRT. Sensitivity analyses restricted to studies adjusting for ypT and ypN yielded consistent results. Future studies should determine whether adding yPNI to established clinicopathological models improves prognostic performance and clinical decision-making.

## Data Availability

The original contributions presented in the study are included in the article/**Supplementary Material**. Further inquiries can be directed to the corresponding author.

## Supplementary material

Appendix 1 Search strategy used in the literature review.

Appendix 2 Multivariable model characteristics and survival endpoint definitions used for effect-size extraction. (A) OS, (B) RFS, (C) DFS, (D) CSS, (E) LRFS, (F) MeFS. Note: OS, overall survival; RFS, recurrence-free survival; DFS, disease-free survival; CSS, cancer-specific survival; LRFS, local recurrence-free survival; MeFS, metastasis-free survival; CI, confidence interval; HR, hazard ratio; PH, proportional hazards; yPNI, post-nCRT perineural invasion; ypN, pathological nodal stage after neoadjuvant treatment; ypT, pathological tumor stage after neoadjuvant treatment.

Appendix 3 PNI definitions reported in the included studies. Note: PNI, perineural invasion; Not specified, no explicit diagnostic definition of PNI was reported in the original study.

Appendix 4 Additional sensitivity analyses of the associations between yPNI and survival outcomes. (A) Sensitivity analysis combining recurrence-free survival and disease-free survival as a single recurrence-related survival outcome; (B) Sensitivity analyses excluding the study by Kim S et al. (2022), which reported an unusually high prevalence of yPNI: (B1) OS and (B2) DFS; (C) Sensitivity analyses excluding studies with potentially overlapping patient populations for (C1) OS and (C2) RFS; (D) Sensitivity analyses restricted to studies with multivariable adjustment for ypT and ypN stage for (D1) OS, (D2) RFS, (D3) DFS, (D4) LRFS and (D5) MeFS. DFS, disease-free survival; OS, overall survival; RFS, recurrence-free survival; yPNI, perineural invasion identified in the surgical specimen after neoadjuvant chemoradiotherapy; ypN, pathological nodal stage after neoadjuvant treatment; ypT, pathological tumor stage after neoadjuvant treatment.

Appendix 5 Trim-and-fill funnel plots assessing the sensitivity of OS outcomes to potential publication bias.
