# Supplementary material for: Prognostic utility of perineural invasion in individuals with rectal cancer receiving neoadjuvant chemoradiotherapy: a meta-analysis
Source: Front Oncol. 2026 Sep 3;16:1845905. doi: 10.3389/fonc.2026.1845905 (PMC13581736; doi:10.3389/fonc.2026.1845905)
Supplement: Supplementary file 1 [file Table1.docx]

Appendix 1 Search strategy used in the literature review

Search strategy of Pubmed.

| No. | Search items |
| --- | --- |
| #1 | "Rectal Neoplasms"[MeSH Terms] |
| #2 | (((((((rectal cancer*[Title/Abstract]) OR (rectal carcinogenesis[Title/Abstract])) OR (rectal malignanc*[Title/Abstract])) OR (Rectal Neoplasm*[Title/Abstract])) OR (Rectal Tumor*[Title/Abstract])) OR (rectum cancer*[Title/Abstract])) OR (rectum malignanc*[Title/Abstract])) OR (Rectum Neoplasm*[Title/Abstract]) |
| #3 | #1 OR #2 |
| #4 | (perineural invasion[Title/Abstract]) OR (perineural tumo* invasion[Title/Abstract]) |
| #5 | #3 and #6 |

Search strategy of Embase.

| No. | Search items |
| --- | --- |
| #1 | 'rectum cancer'/exp |
| #2 | 'rectal cancer*':ab,ti OR 'rectal carcinogenesis':ab,ti OR 'rectal malignanc*':ab,ti OR 'rectal neoplasm*':ab,ti OR 'rectal tumor*':ab,ti OR 'rectum cancer*':ab,ti OR 'rectum malignanc*':ab,ti OR 'rectum neoplasm*':ab,ti |
| #3 | #1 OR #2 |
| #4 | 'perineural invasion'/exp |
| #5 | 'perineural invasion':ab,ti OR 'perineural tumo* invasion':ab,ti |
| #6 | #4 OR #5 |
| #7 | #3 AND #6 |

Search strategy of Cochrane Library.

| No. | Search items |
| --- | --- |
| #1 | MeSH descriptor: [Rectal Neoplasms] explode all trees |
| #2 | (rectal cancer* or rectal carcinogenesis or rectal malignanc* or Rectal Neoplasm* or Rectal Tumor* or rectum cancer* or rectum malignanc* or Rectum Neoplasm*):ti,ab,kw |
| #3 | #1 or #2 |
| #4 | perineural invasion or perineural tumo* invasion |
| #5 | #3 and #4 |

Search strategy of Web of Science.

| No. | Search items |
| --- | --- |
| #1 | rectal cancer* (Topic) OR rectal carcinogenesis (Topic) OR rectal malignanc* (Topic) OR Rectal Neoplasm* (Topic) OR Rectal Tumor* (Topic) OR rectum cancer* (Topic) OR rectum malignanc* (Topic) OR Rectum Neoplasm* (Topic) and Preprint Citation Index (Exclude – Database) and Research Commons (Exclude – Database) |
| #2 | rectal cancer* (Topic) OR rectal carcinogenesis (Topic) OR rectal malignanc* (Topic) OR Rectal Neoplasm* (Topic) OR Rectal Tumor* (Topic) OR rectum cancer* (Topic) OR rectum malignanc* (Topic) OR Rectum Neoplasm* (Topic) and Preprint Citation Index (Exclude – Database) and Research Commons (Exclude – Database) |
| #3 | #2 AND #1 and Preprint Citation Index (Exclude – Database) and Research Commons (Exclude – Database) |

Search strategy of China National Knowledge Infrastructure.

| No. | Search items |
| --- | --- |
| #1 | “直肠癌”[篇关摘] |
| #2 | “神经周围浸润”[篇关摘] |
| #3 | #1 AND #2 |

Search strategy of Wanfang Database.

| No. | Search items |
| --- | --- |
| #1 | “直肠癌”[摘要] |
| #2 | “神经周围浸润”[摘要] |
| #3 | #1 AND #2 |

Search strategy of Chinese Biomedical Literature Database.

| No. | Search items |
| --- | --- |
| #1 | “直肠癌”[摘要:智能] |
| #2 | “神经周围浸润”[摘要:智能] |
| #3 | #1 AND #2 |

Search strategy of VIP Chinese Science and Technology Journal Database.

| No. | Search items |
| --- | --- |
| #1 | “直肠癌”[摘要] |
| #2 | “神经周围浸润”[摘要] |
| #3 | #1 AND #2 |
