# Supplementary material for: Prognostic utility of perineural invasion in individuals with rectal cancer receiving neoadjuvant chemoradiotherapy: a meta-analysis
Source: Front Oncol. 2026 Sep 3;16:1845905. doi: 10.3389/fonc.2026.1845905 (PMC13581736; doi:10.3389/fonc.2026.1845905)
Supplement: Supplementary file 2 [file Table3.docx]

Appendix 3 PNI definitions reported in the included studies

| study | PNI definition |
| --- | --- |
| Sano et al.2025 | Intramural PNI: tumor spread along the Auerbach plexus; extramural PNI: tumor infiltration or extension along nerve fascicles outside the muscularis propria |
| Mülküt et al.2025 | Not specified |
| Luo et al.2025 | Not specified |
| Fan et al.2025 | Not specified |
| Pan et al.2024 | Not specified |
| Naovaset et al.2024 | Not specified |
| Morais et al.2024 | Not specified |
| Hwang et al.2024 | Not specified |
| Cai et al.2024 | Not specified |
| Ulusoy et al.2023 | Not specified |
| Ryu et al.2023 | Not specified |
| Rayan et al.2023 | Not specified |
| Lu et al.2023 | Not specified |
| Liao et al.2023 | Not specified |
| Lim et al.2022 | Not specified |
| Lee,H.G et al.2022 | Not specified |
| Kim,S et al.2022 | Not specified |
| DELİKGÖZ SOYKUT et al.2022 | Not specified |
| Zheng et al.2021 | Not specified |
| Naffouje et al.2021 | Not specified |
| Mei et al.2020 | Tumor cells invading any layer of the nerve sheath or involving >1/3 of the nerve circumference |
| Liu et al.2020 | Not specified |
| Sun et al.2019 | Tumor cells invading any layer of the nerve sheath or surrounding ≥1/3 of the nerve circumference |
| Song et al.2019 | Viable tumor cells within any layer of the nerve sheath or tumor involvement of the nerve circumference in the perineural space |
| Lee,J et al.2019 | Not specified |
| Kim,S.M et al.2019 | Not specified |
| Kim,C.H et al.2019 | Viable tumor cells within any layer of the nerve sheath or in the perineural space involving >33% of the nerve circumference |
| Cho et al.2019 | Not specified |
| Kim et al.2018 | Not specified |
| Vychnevskaia et al.2017 | Not specified |
| Chablani et al.2017 | Presence of neoplastic cells in the perineural space |
| Huh et al.2016 | Not specified |
| Kim et al.2015 | Not specified |
| Huh et al.2015 | Not specified |
| Chung et al.2013 | Not specified |
| Agarwal et al.2013 | Not specified |

PNI, perineural invasion; Not specified, no explicit diagnostic definition of PNI was reported in the original study.
