# Supplementary material for: Prognostic utility of perineural invasion in individuals with rectal cancer receiving neoadjuvant chemoradiotherapy: a meta-analysis
Source: Front Oncol. 2026 Sep 3;16:1845905. doi: 10.3389/fonc.2026.1845905 (PMC13581736; doi:10.3389/fonc.2026.1845905)
Supplement: Supplementary file 3 [file Table4.docx]

Appendix 5 Trim-and-fill funnel plot assessing the sensitivity of OS outcome to potential publication bias.

|  | Egger’s *P* | Pooled effect size using trim-and-fill method, *P* |
| --- | --- | --- |
| OS | 0.024 | 1.809(1.533, 2.136), p < 0.001 |
| DFS | 0.121 |  |
